# *Brucella abortus* Proliferates in Decidualized and Non-Decidualized Human Endometrial Cells Inducing a Proinflammatory Response

**DOI:** 10.3390/pathogens9050369

**Published:** 2020-05-12

**Authors:** Lucía Zavattieri, Mariana C. Ferrero, Iván M. Alonso Paiva, Agustina D. Sotelo, Andrea M. Canellada, Pablo C. Baldi

**Affiliations:** 1 Facultad de Farmacia y Bioquímica, Cátedra de Inmunología, Universidad de Buenos Aires, Buenos Aires 1113, Argentina; mv.lzavattieri@gmail.com (L.Z.); ferrerom@ffyb.uba.ar (M.C.F.); ivan_alonsopaiva@yahoo.com.ar (I.M.A.P.); agustinadsotelo@gmail.com (A.D.S.); acanell@ffyb.uba.ar (A.M.C.); 2 CONICET-Universidad de Buenos Aires, Instituto de Estudios de la Inmunidad Humoral (IDEHU), Buenos Aires 1033, Argentina

**Keywords:** *Brucella abortus*, human endometrial cells, internalization, intracellular replication, decidualization, chemokines, macrophages

## Abstract

*Brucella* spp. have been associated with abortion in humans and animals. Although the mechanisms involved are not well established, it is known that placental *Brucella* infection is accompanied by inflammatory phenomena. The ability of *Brucella abortus* to infect and survive in human endometrial stromal cells (T-HESC cell line) and the cytokine response elicited were evaluated. *B. abortus* was able to infect and proliferate in both non-decidualized and decidualized T-HESC cells. Intracellular proliferation depended on the expression of a functional *virB* operon in the pathogen. *B. abortus* internalization was inhibited by cytochalasin D and to a lower extent by colchicine, but was not affected by monodansylcadaverine. The infection did not induce cytotoxicity and did not alter the decidualization status of cells. *B. abortus* infection elicited the secretion of IL-8 and MCP-1 in either decidualized or non-decidualized T-HESC, a response also induced by heat-killed *B. abortus* and outer membrane vesicles derived from this bacterium. The stimulation of T-HESC with conditioned media from *Brucella*-infected macrophages induced the production of IL-6, MCP-1 and IL-8 in a dose-dependent manner, and this effect was shown to depend on IL-1β and TNF-α. The proinflammatory responses of T-HESC to *B. abortus* and to factors produced by infected macrophages may contribute to the gestational complications of brucellosis.

## 1. Introduction

Human brucellosis, a zoonotic disease mostly caused by *Brucella melitensis*, *B. suis* and *B. abortus*, affects over 500,000 people each year around the world [1]. The infection can be found in several domestic animals (cattle, sheep, goats, pigs, and dogs) and in some wild species. Transmission to humans usually occurs by contact with infected animal tissues and consumption of dairy products.

The clinical manifestations of human brucellosis are usually linked to inflammatory phenomena in the affected organs [2]. Involvement with the reproductive organs is common in animals, which frequently present problems such as abortion and perinatal death. Studies performed in animals have shown that placental *Brucella* infection is accompanied by the infiltration of inflammatory cells [3,4]. The fact that placental inflammatory responses are involved in infection-triggered abortion by several pathogens [5,6,7] suggests that placental inflammation may also have a role in *Brucella*-induced abortion.

Abortion due to *Brucella* infection has been also reported in humans, with an incidence that ranges from 7% to 40% according to different studies [8,9,10]. Among pregnant women who presented with acute brucellosis at a Saudi Arabian hospital, 43% had spontaneous abortion during the first and second trimester, and 2% in the third trimester [11]. In spite of the importance of *Brucella*-related abortion, the pathophysiology of this complication in humans is largely unknown. Recent studies have shown that *Brucella* spp. can infect and replicate in human trophoblasts, and that the infection elicits a proinflammatory response [12,13]. These trophoblastic inflammatory responses may be relevant to the pathogenesis of abortion in human brucellosis. However, the potential of other placental cell populations to contribute to an inflammatory environment during *Brucella* infection has not been explored. 

For several microorganisms that reach the placenta by the hematogenous route, including *Brucella abortus*, in vivo studies in animal models have indicated that the maternal decidua is the initial site of placental colonization [14,15]. Decidualization of the endometrium, a process essential for successful implantation and maintenance of pregnancy, involves progesterone-driven morphological and biochemical changes of fibroblast-like endometrial stromal cells (ESCs) to differentiate into decidual stromal cells (DSCs). These DSCs are characterized by the secretion of prolactin, insulin growth factor-binding protein and several cytokines that act as regulators of the innate immunity [16].

Given the relevance of the decidua as the initial site of placental colonization for several hematogenously spread infections, the ability of decidual cells to respond to pathogens is especially relevant. Primary DSC and ESC cell lines have been shown to express several Toll-like receptors (TLRs) and Nod-like receptors (NLRs), and respond to pathogen-associated molecular patterns (PAMPs) with an enhanced production of matrix metalloproteinases (MMPs) and proinflammatory cytokines including MCP-1, IL-6, IL-8, IL-1β, and CCL5 (RANTES) [17]. At least for group B streptococcal infection the cytokine response of endometrial stromal cells is modulated by decidualization, so that decidualized cells produce IL-6, TNF-α, IL-10, and TGF-β while non-decidualized cells do not [18]. 

In addition to decidual stromal cells, the decidua also contains significant proportions of immune cells, including macrophages, natural killer cells, dendritic cells, and T cells [19]. Early pregnancy is considered to resemble an open wound which requires a strong inflammatory response, thus the first trimester is considered a proinflammatory phase, which turns to an anti-inflammatory phase in the second trimester [20,21]. Although decidual macrophages exhibit an M2 phenotype and exert an immunosuppressive effect on local lymphocyte populations, in the context of local infection they may increase their production of proinflammatory cytokines and contribute to pregnancy disorders [19]. Of note, DSC or ESC have been shown to interact with macrophages in several ways [22,23]. In response to stimulation with lipopolysaccharide (LPS) from *Escherichia coli*, a coculture of ESC and PMA-differentiated THP-1 cells (human monocytes) produced enhanced levels of many cytokines (IL-1β, RANTES, MCP-1, IL-10, TGF-β, MIC-1, G-CSF) as compared to the respective monocultures [24]. Importantly, *B. abortus* is known to survive and replicate in macrophages from several animal species, inducing the secretion of proinflammatory cytokines [25,26,27]. 

The T-HESC cell line, derived from normal primary human ESC by telomerase immortalization, has been widely used to study several aspects of human ESC biology, including infection and cytokine production [23,24,28,29,30,31]. T-HESC are karyotypically, morphologically, and phenotypically similar to the primary parent cells, and after treatment with estradiol and medroxyprogesterone acetate (MPA) display the morphological and biochemical pattern of decidualization [32]. In the present study we evaluated the ability of *Brucella* spp. to infect and survive in decidualized T-HESC, and also assessed the cytokine production induced in these cells by the infection or by their interaction with infected macrophages.

## 2. Results

### 2.1. Brucella abortus Infects and Replicates in Both Decidualized and Non-Decidualized T-HESC Cells

**Figure 1:**
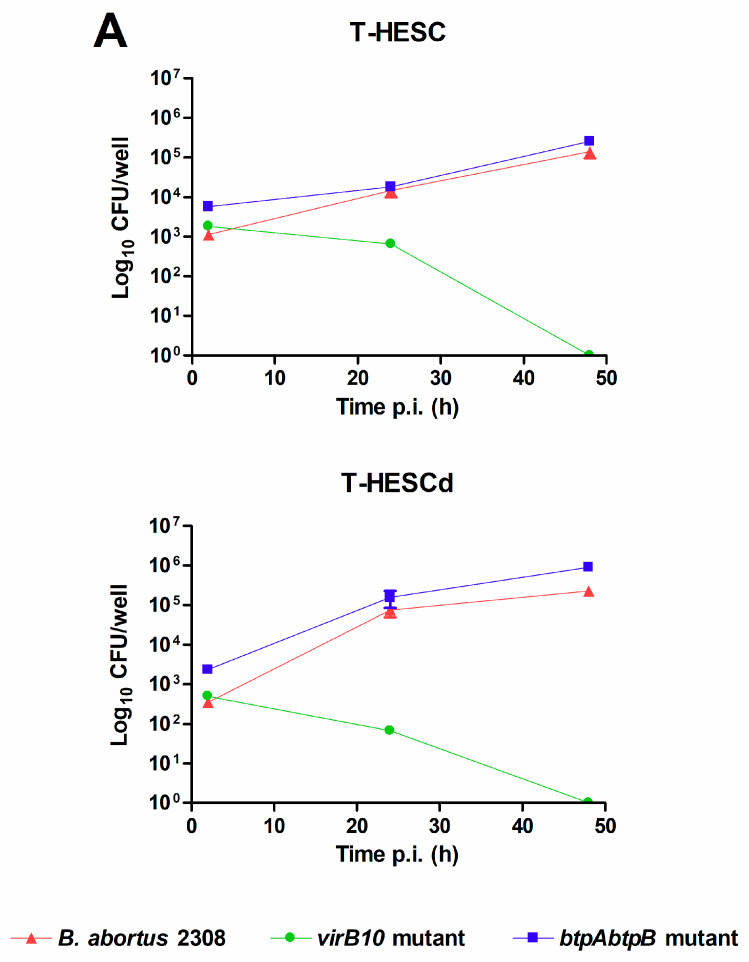

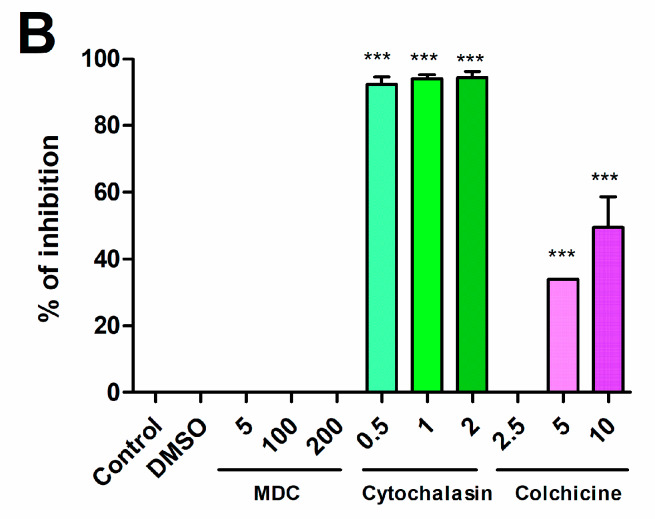
*Brucella abortus* invades and replicates in T-HESC cells. (**A**) Non-decidualized (T-HESC) and decidualized (T-HESCd) endometrial cells were infected with wild type *B. abortus* and two isogenic mutants (*virB10* and *btpAbtpB*), and colony forming unit (CFU) numbers of intracellular bacteria were determined at different times post-infection (p.i.). (**B**). Decidualized T-HESC were pretreated for 1 h with different doses of Colchicine (10, 5, 2.5 µM), Monodansylcadaverine (MDC; 200, 100, 5 μM), Cytochalasin D (2, 1, 0.5 µg/mL), or DMSO (vehicle) before infection with wild type *B. abortus*. Intracellular CFU were determined at 1 h p.i. Results are expressed as mean ± SD from three independent experiments run in duplicates. *** *p* < 0.001 versus control.

Both decidualized and non-decidualized T-HESC endometrial cells were infected with *B. abortus* at a multiplicity of infection (MOI) of 250 bacteria/cell, and colony-forming units (CFU) of intracellular bacteria were determined at different times post-infection (p.i.). As shown in Figure 1, *B. abortus* was able to infect T-HESC cells in both conditions, although the initial number of intracellular bacteria (2 h p.i.) was slightly higher for non-decidualized cells (1125 ± 250 vs. 345 ± 32 CFU/well, mean ± SD). Besides wild type *B. abortus*, two additional strains carrying mutations in genes relevant for virulence were also tested for their capacity to infect and survive in T-HESC cells. These included a mutant lacking the *virB10* gene, widely reported as essential for the intracellular survival and replication of *Brucella* [33,34], and a double mutant lacking *btpA* and *btpB* genes which encode proteins able to interfere with TLR signaling [35,36]. As shown in Figure 1A, both mutant strains were able to infect decidualized and non-decidualized T-HESC at levels similar to the wild type strain. However, the ability to survive and replicate intracellularly differed between the *virB10* mutant and the other two strains. While CFU of intracellular bacteria increased along time for wild type *B. abortus* and the *btpAbtpB* mutant, showing intracellular replication, the CFU of the *virB10* mutant declined at the same time and no viable bacteria were detected in either condition at 48 h p.i. This later result confirmed in endometrial cells the essential role of *virB10* for the intracellular survival of *Brucella*.

Infection experiments were also carried out in the presence of specific inhibitors to examine whether *B. abortus* internalization by T-HESC cells depends on actin polymerization (cytochalasin D), microtubules (colchicine), or clathrin-mediated endocytosis (monodansylcadaverine, MDC). As shown in Figure 1B, *B. abortus* internalization was highly inhibited by cytochalasin D and to a lower extent by colchicine, but was not affected by MDC. 

**Figure 2:**
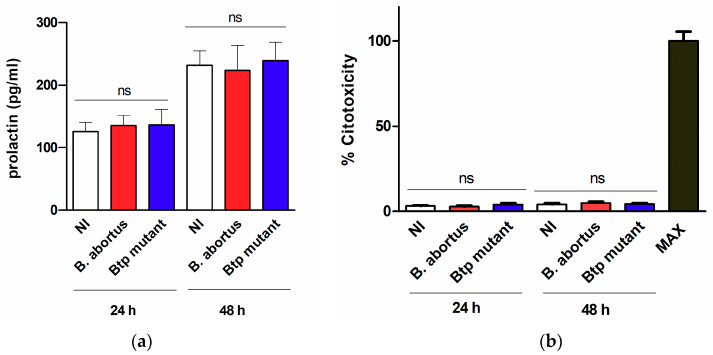
*B. abortus* infection does not induce cytotoxicity or alterations in decidualization in T-HESC cells. Decidualized T-HESC cells were infected or not (NI) with *B. abortus* wild type or its isogenic *btpAbtpB* mutant, and culture supernatants were harvested at 24 and 48 h p.i. to measure the levels of prolactin by ELISA (**a**) and the activity of lactate dehydrogenase (LDH) using a commercial non-radioactive cytotoxicity assay (**b**). In the latter assay, a control of 100% cell lysis (Max) was obtained by hypotonic lysis of the same number of non-infected cells. Results are expressed as mean ± SD from three independent experiments run in duplicates. ns: non-significant versus NI.

To assess whether the infection affected the viability of T-HESC cells or their decidualization status, the levels of lactate dehydrogenase (LDH) and prolactin were measured in culture supernatants of infected cells at 24 and 48 h p.i. and also in non-infected cells cultured in parallel. As shown in Figure 2, the infection with either wild type *B. abortus* or the *btpAbtpB* mutant did not modify the levels of LDH or prolactin as compared to non-infected cells at any time point, showing that it does not induce cytotoxicity or affect the decidualization of cells.

### 2.2. B. abortus Infection Induces the Secretion of Proinflammatory Chemokines in T-HESC Cells

**Figure 3:**
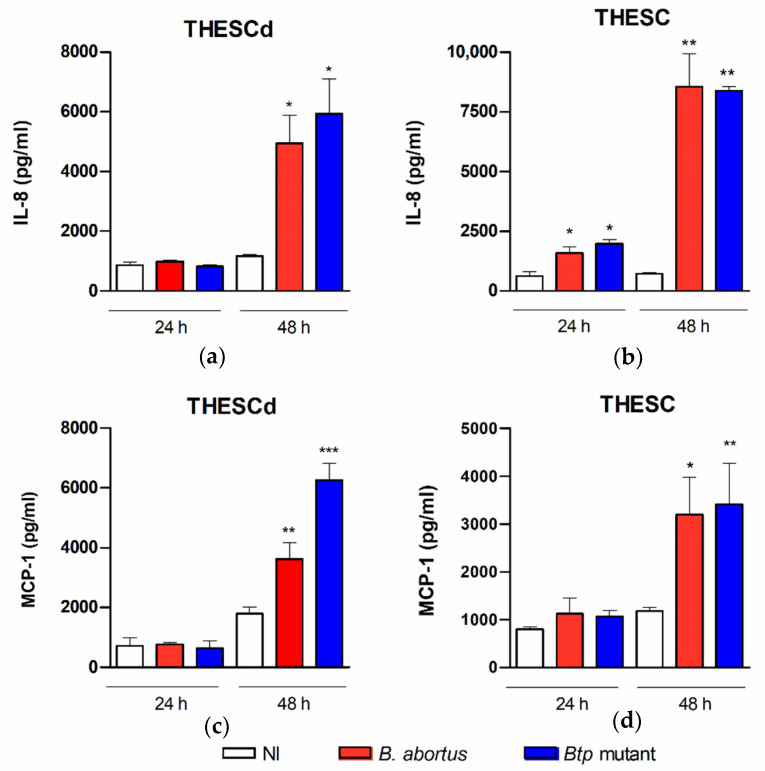
*B. abortus* infection elicits chemokine secretion in T-HESC cells. Decidualized (T-HESCd) (**a**,**c**) and non-decidualized (T-HESC) (**b**,**d**) endometrial cells were infected or not (NI) with wild type *B. abortus* and the *btpAbtpB* mutant, and the levels of IL-8 (**a**,**b**) and MCP-1 (**c**,**d**) were measured by ELISA in culture supernatants harvested at 24 or 48 h p.i. Results are expressed as mean ± SD from three independent experiments run in duplicates. * *p* < 0.05, ** *p* < 0.01, *** *p* < 0.001 versus NI.

As mentioned above, DSC and ESC cell lines express several TLRs and NLRs, and respond to microbial PAMPs with an enhanced production of proinflammatory cytokines, including MCP-1, IL-6, IL-8, IL-1β, and RANTES [17]. To assess the ability of *B. abortus* to induce a proinflammatory response in T-HESC, these cells were infected with the wild type strain and the *btpAbtpB* mutant, and the levels of IL-8 and MCP-1 were measured in culture supernatants. The studies were performed on decidualized and non-decidualized cells to determine whether the proinflammatory response depends on the decidualization status. As shown in Figure 3, the infection with any of the *B. abortus* strains elicited the secretion of both chemokines in either decidualized or non-decidualized T-HESC, and this effect was mostly evident at 48 h p.i. At this time point, IL-8 levels were higher in non-decidualized cells than in decidualized ones (mean, 8539 vs. 4948 pg/mL), whereas no significant difference was found for MCP-1 (mean, 3197 vs. 3621 pg/mL).

**Figure 4:**
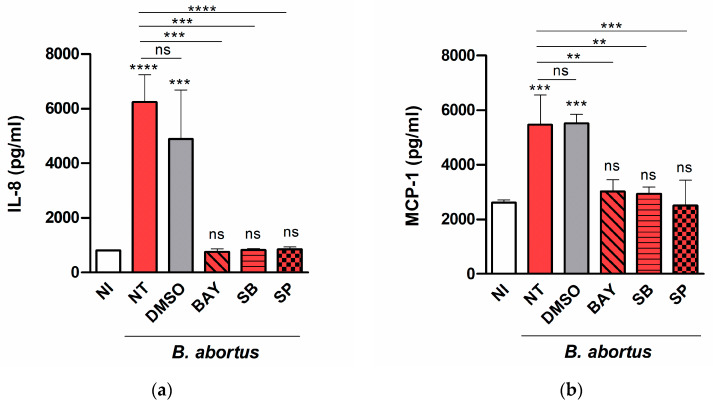
Several signaling pathways are involved in chemokine secretion in *Brucella*-infected T-HESC cells. Decidualized T-HESC cells were treated or not (NT) with SB203580 (SB, p38 MAPK inhibitor), SP600125 (SP, Jnk1/2 inhibitor), BAY 11-7082 (BAY, NF-κB inhibitor), or vehicle (DMSO) for 1 h, and were infected with wild type *B. abortus*. The inhibitors were kept throughout the experiment. At 48 h p.i. culture supernatants were harvested for measuring IL-8 (**a**) and MCP-1 (**b**) by ELISA. Non-treated non-infected cells (NI) served as controls. Results are expressed as mean ± SD from three independent experiments run in duplicates. Asterisks over bars indicate *** *p* < 0.001 or **** *p* < 0.0001 versus NI. Asterisks over lines indicate ** *p* < 0.01, *** *p* < 0.001 or **** *p* < 0.0001 versus NT. ns: non-significant.

To determine which signaling pathways may be involved in the induction of chemokine secretion, decidualized T-HESC cells were treated with SB203580 (p38 MAPK inhibitor), SP600125 (Jnk1/2 inhibitor), BAY 11-7082 (NF-κB inhibitor), or vehicle (dimethyl sulfoxide, DMSO) before and during the infection with *B. abortus*, and IL-8 and MCP-1 were measured as above. As shown in Figure 4, the secretion of both cytokines was not affected significantly by DMSO but was reduced to basal levels by all the inhibitors tested, suggesting that all the signaling pathways (p38, Jnk1/2, and NF-kB) are involved.

**Figure 5:**
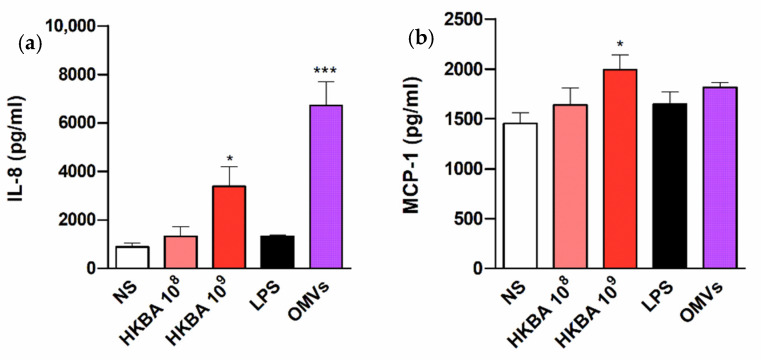
The chemokine response of T-HESC to *B. abortus* does not require bacterial viability. Decidualized T-HESC cells were stimulated or not (NS) with two doses (10^8^ and 10^9^ CFU/mL) of heat-killed *B. abortus* (HKBA), or with lipopolysaccharide (LPS) or outer membrane vesicles (OMVs) from this bacterium, and IL-8 (**a**) and MCP-1 (**b**) levels were measured in culture supernatants at 48 h post-stimulation. Results are expressed as mean ± SD from three independent experiments run in duplicates. * *p* < 0.05, *** *p* < 0.001 versus NS.

Given the ability of *B. abortus* infection to induce the secretion of IL-8 and MCP-1 in T-HESC cells, experiments were carried out to determine whether such responses can be also elicited by stimulation with *B. abortus* antigens or, conversely, depend on bacterial viability. For this purpose, cells were stimulated with either heat-killed *B. abortus* (HKBA), or LPS or outer membrane vesicles (OMVs) from this bacterium, and chemokine levels were measured at 48 h p.i. As shown in Figure 5, HKBA (at 10^9^ CFU/mL) elicited IL-8 and MCP-1 secretion by T-HESC cells, albeit at lower levels than those attained by the infection. In addition, IL-8 secretion was significantly induced by *B. abortus* OMVs. These results show that the induction of chemokines in these cells does not depend on *Brucella* viability.

### 2.3. Factors Produced by Brucella-Infected Macrophages Stimulate Proinflammatory Responses in Decidualized T-HESC Cells

**Figure 6:**
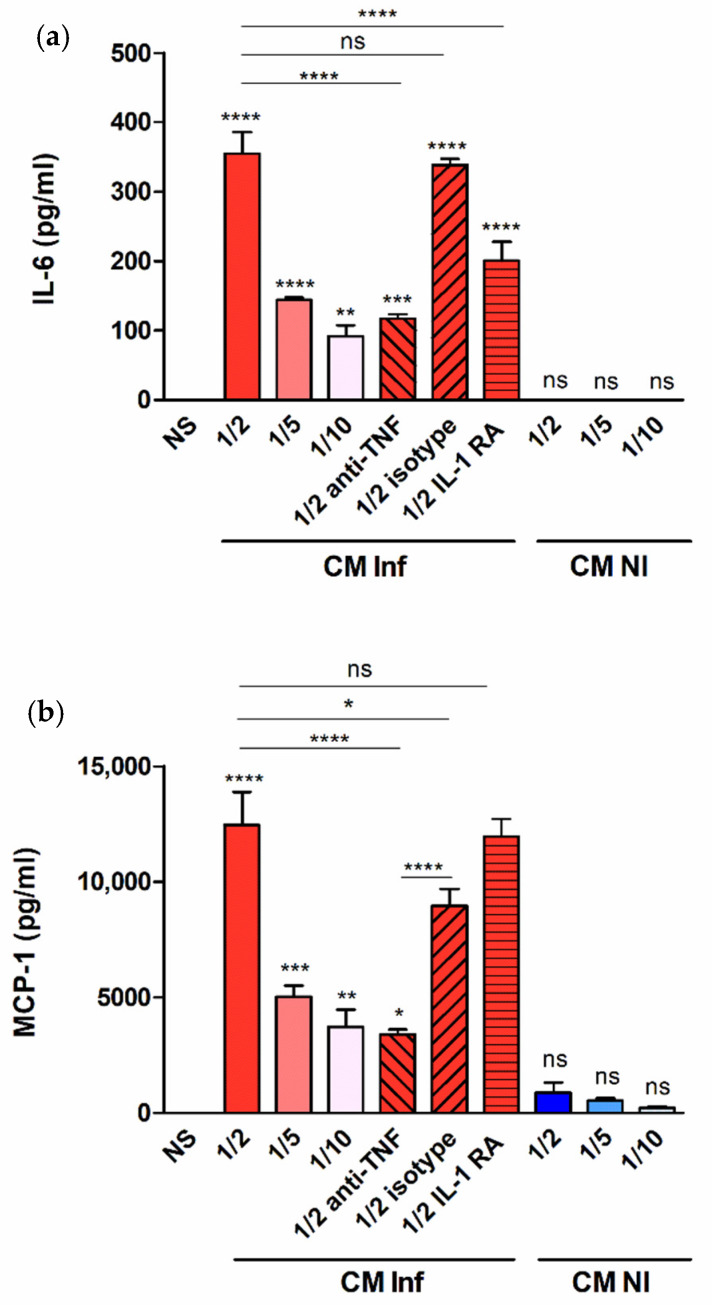

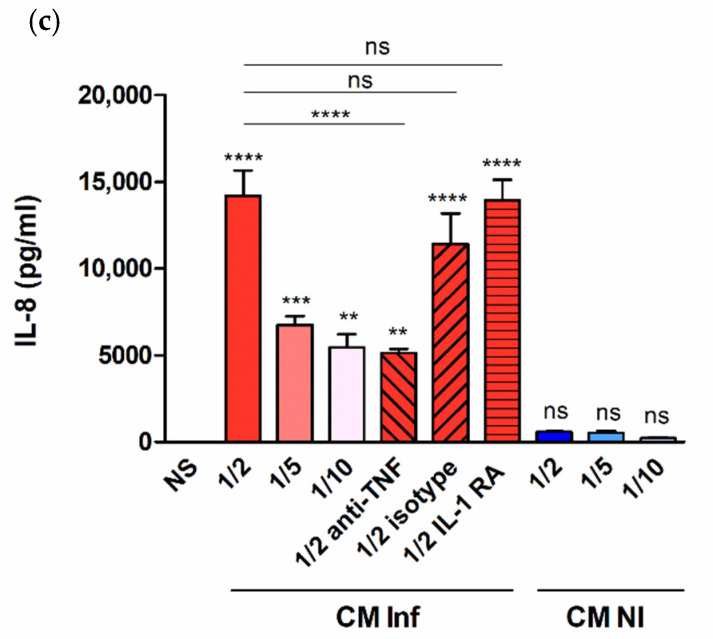
Factors produced by *B. abortus*–infected macrophages stimulate cytokine production by endometrial cells. Decidualized T-HESC cells were stimulated or not (NS) with conditioned media from *B. abortus*-infected macrophages (CM Inf) or from uninfected macrophages (CM NI) at different dilutions (1/2, 1/5 or 1/10), and 24 h later culture supernatants were harvested to measure IL-6 (**a**), MCP-1 (**b**) and IL-8 (**c**) levels. In parallel experiments, CM Inf was preincubated with a TNF-α neutralizing antibody or an isotype control before addition to cells, or T-HESC were preincubated with the natural antagonist of the IL-1 receptor (IL-1Ra) before stimulation with CM Inf, and cytokine levels were measured as described. Results are expressed as mean ± SD from three independent experiments run in duplicates. * *p* < 0.05, ** *p* < 0.01, *** *p* < 0.001, **** *p* < 0.0001, ns: non-significant. Asterisks over bars indicate differences versus NS.

The results shown above demonstrate that decidualized T-HESC produce proinflammatory mediators in response to infection with *B. abortus* or stimulation with its antigens. In the context of infection in the pregnant uterus, however, endometrial cells may also receive stimulation by factors produced by adjacent infected macrophages [22,23]. To model this scenario in vitro, decidualized T-HESC cells were stimulated with conditioned media (CM) from *B. abortus*-infected macrophages and the levels of proinflammatory cytokines were measured in culture supernatants 24 h later. The preexisting levels of these cytokines in the CM were subtracted in order to calculate the secretion specifically induced by the stimulation. As shown in Figure 6, stimulation with CM from *Brucella*-infected macrophages induced the production of IL-6, MCP-1, and IL-8 in a dose-dependent manner (higher secretion for stimulation with CM diluted at 1/2). No significant secretion of any of these cytokines was induced by stimulation with CM from non-infected monocytes. Previous similar studies on the stimulation of other non-phagocytic cells have shown that IL-1β and TNF-α are involved in the inducing effect of CM from *Brucella*-infected macrophages. To test whether these cytokines are also involved in the stimulation of IL-6, IL-8, and MCP-1 in decidualized T-HESC cells, experiments were performed in which CM were preincubated with a TNF-neutralizing antibody or T-HESC were preincubated with the natural antagonist of the IL-1 receptor (IL-1Ra). As shown in Figure 6, the stimulating effect of the CM on the secretion of IL-6 was significantly reduced by both pretreatments, implying that both TNF-α and IL-1β are involved. For MCP-1 and IL-8, in contrast, only the preincubation with the anti-TNF antibody produced a significant reduction. Although the isotype control also produced a significant reduction of MCP-1 levels, the reducing effect of the specific anti-TNF antibody was much more pronounced. In summary, TNF-α and/or IL-1β are involved in the ability of CM from *Brucella*-infected macrophages to stimulate the production of proinflammatory cytokines by decidualized T-HESC.

## 3. Discussion

*Brucella* infections have been associated with abortion in both humans and animals. Although the pathophysiology of this complication has not been completely elucidated, the inflammatory phenomena observed in the affected placenta [3,4] suggest that, as with other pathogens causing abortion, placental inflammation may have a role in *Brucella*-induced abortion. As *Brucella* can reach the placenta by the hematogenous route, the maternal decidua is probably the initial site of placental colonization [14,15]. Given the known ability of decidual cells to respond to microbial PAMPs with an enhanced production of proinflammatory cytokines, and the known deleterious effect of placental inflammation on gestation, we decided to assess the ability of *Brucella* spp. to colonize decidualized stromal endometrial cells (T-HESC) and to induce the production of proinflammatory cytokines. 

As shown here, *B. abortus* was able to infect both decidualized and non-decidualized T-HESC cells, although the initial number of intracellular bacteria was slightly higher for non-decidualized cells. This may relate to the fact that decidualized cells form an organized layer thus exposing less membrane surface to the environment. In addition, the pathogen was able to survive and replicate inside these cells. These findings are in line with the reported ability of *B. abortus* for intracellular replication in several phagocytic and non-phagocytic cells, including macrophages, epithelial cells and trophoblasts [13,25,37]. It has been widely demonstrated that this ability for intracellular survival in different cell types depends on the expression of a type IV secretion system encoded by the *virB* operon, which allows *Brucella* to modulate phagosome-lysosome fusion [33,34]. In line with this, we found that a *B. abortus* mutant lacking the *virB10* gene was unable to survive and replicate inside decidualized and non-decidualized T-HESC despite a similar ability of invasion compared to the wild type strain. In contrast, a mutant lacking the genes for the BtpA and BtpB proteins that interfere with TLR signaling exhibited invasion and replication abilities similar to the wild type strain. Importantly, *B. abortus* infection did not induce cytotoxicity, nor did it affect the decidualization status of cells, suggesting that the decidua might sustain the infection in affected individuals.

The mechanisms for *Brucella* invasion of non-phagocytic cells may vary according to the cell type considered. Whereas actin polymerization and microtubules have been involved in many cells [37], internalization in Vero cells does not depend on microtubules but depends on clathrin-mediated endocytosis [38]. The requirements for invasion of endometrial cells have not been reported. We found that *B. abortus* internalization was inhibited by cytochalasin D and to a lower extent by colchicine, which inhibit actin polymerization and microtubule formation, respectively. In contrast, internalization was not affected by MDC, an inhibitor of clathrin-mediated endocytosis.

As mentioned previously, placental *Brucella* infection is accompanied by the infiltration of inflammatory cells [3,4], which suggests that placental inflammation may have a role in *Brucella*-induced abortion as it does in abortion triggered by other pathogens. Our results show that *B. abortus* infection elicits the secretion of IL-8 and MCP-1 in either decidualized or non-decidualized T-HESC cells. IL-8 levels were higher in non-decidualized cells than in decidualized ones, whereas no significant difference was found for MCP-1. The higher production of IL-8 in non-decidualized cells may relate to the higher number of intracellular bacteria found in this condition as compared to decidualized cells, a downmodulating effect of decidualization on IL-8 production [39], or both. Nonetheless, these results suggest that, although the decidualization status may influence the levels of some proinflammatory mediators, decidualized endometrial cells are capable of mediating a proinflammatory response to *B. abortus*. A few previous studies have shown that *Brucella* BtpA and BtpB proteins, which contain TIR motifs and can thus modulate TLR signaling, can reduce cytokine production in dendritic cells in vitro (IL-12, TNF-α) and in lung tissues in vivo (IL-12, CXCL-1, MCP-1) [35,36]. However, the potential modulating role of these proteins in *Brucella*-infected endometrial cells was unknown. At variance with those previous studies, we did not detect significant differences in the levels of the two chemokines here evaluated (IL-8 and MCP-1) between T-HESC infected with the wild type *B. abortus* strain or the *btpAbtpB* mutant strain. These results agree with those reported for the same chemokines in *Brucella*-infected human trophoblasts [12], and add support to the hypothesis that the immune responses of professional phagocytes are more influenced by the action of Btp proteins than those of non-phagocytic cells. 

The secretion of both cytokines was reduced to basal levels by all the inhibitors tested, suggesting that all the signaling pathways (p38, Jnk1/2, and NF-kB) are involved. In line with these findings, previous studies have shown that several signaling pathways are involved in cytokine production by different cell types in response to *B. abortus*. For example, CCL20 secretion by human bronchial epithelial cells depends on p38, Jnk1/2, Erk1/2, and NF-kB [40], whereas in murine astrocytes TNF-α secretion depends on p38 and Erk1/2 signaling pathways [41]. 

Previous studies in several non-phagocytic cells have shown that not only live *B. abortus* but also some of its antigens can elicit the production of proinflammatory cytokines [42,43,44]. In line with these reports, we found that HKBA and OMVs from *B. abortus* elicit IL-8 and/or MCP-1 secretion in T-HESC cells. Obviously, these findings imply that the induction of chemokines in these cells does not depend on *Brucella* viability. At variance with HKBA and OMVs, *B. abortus* LPS did not elicit the production of the chemokines analyzed. This result is in line with previous studies in other cell types, which demonstrated that *B. abortus* LPS is a poor inducer of proinflammatory responses [42,43,44,45]. In contrast, most inflammatory responses are triggered by outer membrane lipoproteins, which induce TLR2 signaling [45].

As shown in this study, decidualized T-HESC produce proinflammatory mediators, including MCP-1, in response to infection with *B. abortus* or stimulation with its antigens. However, the decidua contains not only DSC but also a significant proportion of macrophages [19], with which DSC can establish reciprocal interactions [22,23]. In addition, the number of decidual macrophages could eventually augment in the context of locally increased MCP-1 levels induced by an infectious process. Therefore, it can be speculated that, during *B. abortus* infection in the pregnant uterus, endometrial cells may respond not only to the stimulus of bacterial antigens but also to stimulation by factors produced by adjacent *Brucella*-infected macrophages. In support of this hypothesis, we found that the stimulation of decidualized T-HESC with CM from *B. abortus*-infected macrophages induced the production of IL-6, MCP-1, and IL-8 in a dose-dependent manner, a phenomenon not produced by stimulation with CM from non-infected monocytes. Additional studies using specific blocking agents revealed that IL-6 induction by CM is mediated by TNF-α and IL-1β, whereas the induction of MCP-1 and IL-8 is mediated by TNF-α. These findings are similar to those reported for the interaction between *Brucella*-infected macrophages and human trophoblasts [12].

Overall, these results suggest a possible scenario in which DSC produce IL-6 and chemoattractants for monocytes/macrophages in response to *B. abortus* infection and/or in response to cytokines produced by *Brucella*-infected placental macrophages. Reciprocal stimulations between DSC and phagocytes may amplify these phenomena. These interactions may be long-lasting due to the ability of *Brucella* to survive and replicate within macrophages and DSC. Altogether, these proinflammatory responses may contribute to the gestational complications of brucellosis.

## 4. Materials and Methods

### 4.1. Reagents

LPS from *Brucella abortus* 2308 was provided by Ignacio Moriyón (University of Navarra, Pamplona, Spain). The purity and the characteristics of this preparation have been published previously [46].

### 4.2. Cell lines

A human endometrial stromal cell line (T-HESC) was kindly provided Dr. Andrea Randi (Human Biochemistry Department, School of Medicine, University of Buenos Aires). This cell line was derived from normal stromal cells obtained from an adult patient subjected to hysterectomy, and conserved the characteristics of the regular endometrial cells [32]. The line was obtained by immortalization by transfection of telomerase (hTERT) using a retroviral system, and expressed puromycin resistance genes. Cells were maintained in DMEM-F12 supplemented with 10% FCS, 50 U/mL penicillin, 50 µg/mL streptomycin, 2 mM glutamine and 500 ng/mL puromycin. Decidualization was achieved following published procedures [47]. Briefly, T-HESC (5 × 10^4^ cells/well) were treated with medroxyprogesterone acetate (MPA, 10^−7^ M) and dibutyryl cAMP (0.5 mM) for 8 days, changing the culture media every 48 h. Decidualization was evaluated by morphology and by prolactin levels measured by sandwich ELISA (R&D Systems). For infection assays, cells were cultured for 24 h in antibiotic-free culture medium.

### 4.3. Monocyte Isolation and Macrophage Differentiation

Peripheral blood samples were obtained from healthy volunteers after approval by the Ethics Committee of the School of Pharmacy and Biochemistry (Approval 2194/17). Written informed consent was obtained from all volunteers. Human monocytes were isolated by standard procedures. Briefly, whole blood diluted with sterile phosphate-buffered saline (PBS) was carefully layered on Ficoll-Paque (density: 1.077 g/mL) and centrifuged at 400× *g* for 30 min. The layer containing peripheral blood mononuclear cells was carefully removed by pipetting and washed with PBS by centrifugation at 250× *g* for 10 min. The pellet was resuspended in RPMI 1640 medium supplemented with 1 mM glutamine and was incubated for 2 h in 24-well plates. After washing with sterile PBS to eliminate nonadherent cells, RPMI medium supplemented with 10% sera from the same donors and antibiotics (100 U/mL penicillin and 100 µg/mL streptomycin) was added to the adherent cells. Cells were incubated at 37 °C in a 5% CO_2_ atmosphere for 7 days for in vitro macrophagic differentiation [48]. Antibiotics were removed 24 h prior to infection.

### 4.4. Bacterial Strains and Growth Conditions

*B. abortus* 2308 (wild type strain), its isogenic *btpAbtpB* double mutant and *virB10* polar mutant were obtained from our collection. The strains were grown in tryptic soy broth at 37 °C with agitation. After two washes with sterile PBS, bacterial inocula were adjusted to the desired concentration in sterile PBS based on optical density readings. An aliquot of each suspension was plated on tryptic soy agar (TSA) and incubated at 37 °C to determine the actual concentration of colony-forming units (CFU) in the inocula. Cells were inoculated with *B. abortus* 2308 at an MOI of 200 and the plates were centrifuged for 10 min at 1200 rpm at room temperature. After 2 h, culture medium was removed and replaced with medium containing gentamicin and streptomycin. All live *Brucella* manipulations were performed in biosafety level 3 facilities. To prepare HKBA, bacteria were washed in sterile PBS, heat killed at 70 °C for 30 min, aliquoted, and stored at −80 °C until use. The absence of bacterial viability was checked by plating on TSA.

### 4.5. Isolation of Outer Membrane Vesicles

OMVs from *B. abortus* 2308 were obtained essentially as described previously [49]. Briefly, bacteria were grown as described above, harvested by centrifugation and washed twice in sterile PBS. The pellet was resuspended in Gerhardt-Wilson minimal medium at an OD_600 nm_ of 0.1 and cultured for 72 h (early stationary phase of growth). The culture was centrifuged, and the cell-free supernatant was filter-sterilized. The filtrate was centrifuged at 15,000× *g* for 5 h at 4 °C. The pellets containing the OMVs were resuspended in PBS, and protein concentration was measured by the bicinchoninic acid assay (Pierce). The presence of OMVs was corroborated by electron microscopy. OMVs were stored at −20 °C until use.

### 4.6. Stimulation of T-HESC Cells with Brucella antigens

Decidualized T-HESC cells (5 × 10^4^ cells/well) were stimulated with LPS from *B. abortus* (1 µg/mL), OMVs (1 µg/mL of protein), or HKBA (10^9^ or 10^8^ CFU/mL). Cells were cultured at 37 °C in a 5% CO_2_ atmosphere, and supernatants were collected 48 h after stimulation for chemokine measurement. 

### 4.7. Cellular Infections

Decidualized and non-decidualized T-HESC cells were infected with *B. abortus* 2308 at MOI of 250 bacteria/cell. Monocyte-derived macrophages were infected at MOI 100 bacteria/cell in culture medium containing no antibiotics. After dispensing the bacterial suspension, the plates were centrifuged (10 min at 400× *g*) and then incubated for 2 h at 37 °C in a 5% CO_2_ atmosphere. Non-internalized bacteria were eliminated by several washes with medium alone followed by incubation in medium supplemented with 100 µg/mL gentamicin and 50 µg/mL streptomycin. After that, cells were washed and then incubated with culture medium without antibiotics. At different times post-infection (2, 24 or 48 h) culture supernatants were harvested for cytokine measurement, while the cells were washed with sterile PBS and lysed with 0.2% Triton X-100. Serial dilutions of the lysates were plated on TSA to enumerate intracellular CFU. In addition, the levels of prolactin were measured in culture supernatants as described above to assess the impact of infection on the decidualization status of the cells, and the levels of LDH were measured to assess cytotoxicity. 

### 4.8. Evaluation of Cytotoxicity 

To analyze the effect of infection on cell integrity, the release of lactate dehydrogenase (LDH) from infected T-HESC cells was determined. LDH activity was determined using the CytotTox 96 Non-Radiactive Cytotoxicity Assay (Promega, USA) in culture supernatants obtained at 24 and 48 h p.i. Results were expressed as the ratio between LDH levels measured in the samples (infected or non-infected cultures) and those corresponding to a 100% cell lysis (obtained by hypotonic lysis of the same number of cells).

### 4.9. Internalization Pathways

To assess the role of microtubules, actin or clathrin in *B. abortus* internalization, decidualized T-HESC were pretreated for 1 h with different doses of colchicine (10, 5, 2.5 µM, Sigma), monodansylcadaverine (MDC; 200, 100, 5 μM) or cytochalasin D (2, 1, 0.5 µg/mL, Sigma) and were later infected as described above but in the presence of these inhibitors. MDC and cytochalasin were solubilized in dimethyl sulfoxide (DMSO), and in all the experiments control cells were incubated without inhibitor or with DMSO for the same period as treated cells. Intracellular CFU were determined at 2 h p.i. as described above.

### 4.10. Stimulation of T-HESC with Conditioned Media (CM) from Brucella-Infected Macrophages

CM from macrophages infected with *B. abortus* 2308 (MOI 100) were harvested at 24 h p.i., filter-sterilized and used to stimulate noninfected decidualized T-HESC cells. After 24 and 48 h, supernatants from stimulated cultures were harvested to measure cytokines. The preexisting levels of cytokines in the CM were subtracted in order to calculate the secretion specifically induced by the stimulation. To determine if TNF-α might be involved in the stimulating effects of CM, in some experiments CM were preincubated for 1 h at 37 °C with a neutralizing monoclonal antibody against TNF-α or its isotype control (both from BD Pharmingen) before being transferred to T-HESC cultures. Alternatively, to determine the role of IL-1 β in the stimulating effect, decidualized T-HESC cells were preincubated with the IL-1β receptor antagonist IL-1Ra (R&D Systems) for 1 h at 37 °C before stimulation with CM from infected macrophages.

### 4.11. Inhibition of Signaling Pathways

To examine the signaling pathways involved in cytokine secretion, decidualized T-HESC cells were pretreated with 10µM SB203580 (p38 MAPK inhibitor, Gibco), 10 µM SP600125 (Jnk1/2 inhibitor, Sigma), 2.5 µM BAY11-7082 (NF-κB inhibitor, Sigma) or vehicle (DMSO). These reagents were added one hour before infection with *B. abortus* and were kept throughout the experiment (48 h). Cell viability after incubation with these inhibitors was higher than 90%, as assessed by staining with trypan blue. 

### 4.12. Measurement of Cytokines and Chemokines

Levels of human IL-6, IL-8, MCP-1, and TNF-α were measured in culture supernatants by sandwich ELISA, using paired cytokine-specific monoclonal antibodies according to the manufacturer’s instructions (BD Pharmingen).

### 4.13. Statistical Analysis

Each experiment was performed in duplicates on three independent occasions. The values obtained are presented as the mean ± SD. Statistical analysis was performed with one-way ANOVA, followed by Post Hoc Tukey’s Test or Dunnett’s Test using GraphPad Prism 6.0 software.
